# Niemann-Pick type C1 protein regulates epidermal growth factor receptor stability in hepatic cells and facilitates the early steps of Hepatitis B virus infection

**DOI:** 10.3389/fcimb.2026.1905331

**Published:** 2026-08-20

**Authors:** Stefania Ariadna Stefan-Buzatoiu, Petruta-Ramona Alexandru Flintoaca, Mihaela-Olivia Dobrica, Stefana-Maria Petrescu, Norica Branza-Nichita

**Affiliations:** 1 Department of Viral Glycoproteins, Institute of Biochemistry of the Romanian Academy, Bucharest, Romania; 2 Department of Molecular Cell Biology, Institute of Biochemistry of the Romanian Academy, Bucharest, Romania

**Keywords:** cholesterol, EGFR, HBV infection, lipid metabolism, NPC1

## Abstract

**Background:**

Chronic Hepatitis B virus (HBV) infection affects more than 250 million individuals worldwide, causing up to 1 million deaths yearly. Despite the significant disease burden, no curative therapy against HBV is currently available. Accumulating evidence indicate a strong and complex relationship between HBV and the host-cell lipid metabolism. HBV hijacks host lipoprotein transport pathways to gain access to hepatocytes and uses cellular cholesterol and sphingolipids for morphogenesis, while viral replication induces diverse and profound alterations of the hepatic lipid metabolism driving disease progression and tumorigenesis. As a key regulator of the cellular cholesterol homeostasis, the Niemann-Pick C1 (NPC1) protein has increasingly been associated with a wide range of pathologies, including viral infections.

**Methods:**

In this study, we investigated HBV entry, replication, viral particle envelopment and secretion in cells depleted of NPC1 expression or treated with various FDA-approved NPC1 inhibitors.

**Results:**

Our work revealed that both genetic depletion and pharmacological inhibition of NPC1 impaired the early steps of HBV infection while significantly reducing the level and intracellular distribution of the HBV co-receptor, the Epidermal Growth Factor Receptor (EGFR).

**Discussion:**

Mechanistically, NPC1 inhibition promoted EGFR depletion from the plasma membrane by enhancing the endocytic trafficking and degradation of the receptor within the endosomal-lysosomal compartment of hepatic cells. Considering the crucial roles of EGFR in tumorigenesis across multiple cancer types, our study promotes the newly identified NPC1-EGFR regulatory axis as a promising dual target for the development of antiviral and anticancer therapies.

## 1 Introduction

Despite substantial progress being made in treatment of Hepatitis B virus (HBV) infection, more than 250 million chronically-infected people worldwide remain at high risk to develop advanced liver disease and hepatocellular carcinoma (Hepatitis B). The ultimate goal to achieve a curative treatment of chronic HBV infection has not yet been met due to the difficulty to eradicate the covalently closed circular DNA (cccDNA) nuclear replication form from infected hepatocytes. Therefore, new research strategies rather focus on attaining a functional cure, i.e., sustained undetectable blood levels of hepatitis B surface antigen (HBsAg) and HBV DNA after therapy, by targeting HBV-host cell interactions and/or the activation of the host antiviral immune response (Lim et al., 2023; Lok, 2024). Since initial establishment of the cccDNA reservoir relies on *de novo* infection, targeting viral entry would be a sensible approach to prevent HBV spread in the liver and progression of the disease.

HBV infection is initiated by attachment of the preS1 region of the large (L) envelope protein to sodium taurocholate co-transporting polypeptide (NTCP), a liver-specific, transmembrane bile acid transporter expressed on the hepatocyte surface (Yan et al., 2012). HBV internalization is further mediated by NTCP binding to the epidermal growth factor receptor (EGFR) that facilitates endocytosis of the virus-receptor complex and transport through the endosomal pathway (Iwamoto et al., 2019). Interestingly, unlike in the case of other viruses, EGFR downstream signaling plays no role in HBV infection (Iwamoto et al., 2020).

Stability and function of transmembrane receptors critically depend on membrane lipid composition. Lipids control the physio-chemical properties of the membranes, consequently affecting receptor–ligand interactions, receptor clustering, signaling, endocytosis and intracellular trafficking (van Meer et al., 2008). As a major constituent of membrane lipids, cholesterol regulates these processes by altering membrane fluidity, curvature and packaging of the lipid bilayer or by directly binding to receptor proteins (Subczynski et al., 2017; Pohnl et al., 2023). Owing it to these multiple regulatory mechanisms, cholesterol has been involved in the life-cycle of a variety of enveloped viruses, including HBV (Ahmad et al., 2023). Infectivity of HBV particles produced in cells treated with cholesterol-lowering drugs is significantly reduced (Bremer et al., 2009), while cell depletion of cholesterol affects the topology of the HBV-L protein at the endoplasmic reticulum (ER) membrane resulting in impaired envelopment of viral particles (Dorobantu et al., 2011).

Normal distribution of lipids to various cellular compartments relies on the activity of the late endosome/lysosome-residing Niemann-Pick C1 (NPC1), a cholesterol binding transmembrane protein that controls the lysosomal export of cholesterol derived from the endocytosed low density lipoproteins (Infante et al., 2008; Kwon et al., 2009). Mutations in the NPC1 encoding gene result in accumulation of cholesterol and sphingolipids within the late endosomes/lysosomes, impairment of the endocytic sorting and recycling pathways causing the Niemann-Pick disease, a rare and severe autosomal pathology affecting the brain, lung, liver and other tissues (Pentchev et al., 1985; Fiorenza et al., 2018).

Interestingly, NPC1 is a bona fide receptor for Ebola virus, Marburg virus and other filoviruses, independent of its function in cholesterol transport (Carette et al., 2011). More recently, NPC1 has been directly involved in SARS-CoV-2 entry, when the virus engages the endocytic pathway for internalization (Khan et al., 2024).

In this study we showed that NPC1 inhibition or genetic downregulation of the protein in various HBV infection/replication - permissive cell lines, resulted in significant inhibition of the early-steps of the HBV life-cycle while neither viral replication nor particle morphogenesis and secretion were affected. At the time of our manuscript preparation for submission, another study has been published, very well documenting a role for NPC1 in supporting the HBV endosomal release, mediated by the NPC1 function as a cholesterol transporter (He et al., 2026). Consistent with the reported role of NPC1 at early stages of HBV infection, our work also indicates a significant change of EGFR expression and intracellular distribution in NPC1 depleted/inhibited cells. Mechanistically, NPC1 inhibition promoted EGFR depletion from the plasma membrane by enhancing the endocytic trafficking and degradation of the receptor within the endosomal-lysosomal compartment of hepatic cells, while NTCP remained unaffected. Considering the crucial roles played by EGFR not only in viral infections but also in tumor development, our findings unravel a novel mechanism promoting NPC1 as a dual therapeutic target for broad spectrum antiviral and antitumoral drugs.

## 2 Materials and methods

### 2.1 Cell culture

HepG2-hNTCP cells, kindly provided by Prof. Stephan Urban (Heidelberg University Hospital), were maintained in Dulbecco’s modified Eagle medium (DMEM; Gibco/Invitrogen) supplemented with 10% fetal bovine serum (FBS) (Life Technologies), 1% non-essential amino acids (Sigma-Aldrich) and 5 μg/mL puromycin (Thermo Fisher Scientific). HepaRG cells (a kind gift from Dr. David Durantel, INSERM U871, Lyon, France) were cultured in William’s E Medium GlutaMAX supplemented with 10% FBS, 1% penicillin–streptomycin (Gibco), 5 μg/mL insulin (Sigma-Aldrich), 1% non-essential amino acids (Sigma-Aldrich) and 5 × 10^-5^ M hydrocortisone hemisuccinate (Sigma-Aldrich) following previously described conditions (Macovei et al., 2013). Huh7 cells were grown in Dulbecco’s Modified Eagle Medium (DMEM) supplemented with GlutaMAX™-I (Life Technologies), 10% FBS, 1% non-essential amino acids, and 1% penicillin/streptomycin. HepG2 2.2.15 cells, which stably produce infectious HBV particles (kindly provided by Dr. David Durantel, INSERM U871, Lyon, France), were cultured as previously described (Dorobantu et al., 2011). All cell lines were maintained at 37 °C in a humidified atmosphere containing 5% CO2. Cell cultures were routinely screened for mycoplasma contamination by PCR using the Venor^®^ GeM OneStep kit (Minerva Biolabs).

### 2.2 Antibodies

For Western blot analyses, the following primary antibodies were used: rabbit anti-sodium taurocholate co-transporting polypeptide (NTCP; Santa Cruz Biotechnology H-42, sc-98484, 1:500), rabbit anti-Niemann-Pick C1 (NPC1; Abcam, ab134113, 1:1000), mouse anti-EGFR (A-10, Santa Cruz Biotechnology, sc-373746, 1:300), rabbit anti-calnexin (Abcam, ab22595, 1:10000) and rat anti-HA (Roche, 11867423001, 1:1000). Secondary antibodies used for Western blot included HRP-conjugated anti-rabbit IgG (Cell Signaling Technology, 7074S, 1:8000), anti-mouse IgG (Cell Signaling Technology, 7076P2, 1:8000) and anti-rat IgG (Jackson ImmunoResearch, 712-035-150, 1:10000). For immunofluorescence microscopy, the following primary antibodies were used: mouse anti-EGFR (A-10, Santa Cruz Biotechnology, sc-373746, 1:200), rabbit anti-NTCP (Invitrogen, PA5-80001, 1:100), mouse anti-β-catenin (E-5, Santa Cruz Biotechnology, sc-7963, 1:100), rabbit anti-CD63 (Lamp3) (H-193, Santa Cruz Biotechnology, sc-15363, 1:100). Samples were subsequently incubated with Alexa Fluor 488-conjugated donkey anti-rabbit IgG (Invitrogen, A-21206, 1:800), Alexa Fluor 488-conjugated donkey anti-mouse IgG (Invitrogen, A-21202, 1:800) and Alexa Fluor 594-conjugated goat anti-mouse IgG secondary antibodies (Invitrogen, A-11205, 1:800). For immunoprecipitation experiments, mouse anti-HBV core (Invitrogen, MA1-7607, 1:400) and mouse anti-HepB preS1 (AP2, Santa Cruz Biotechology, sc-57762, 1:400) were used. For In-Cell ELISA, primary antibodies against NTCP (Invitrogen, PA5-80001, 1:100) and EGFR (Santa Cruz Biotechnology, sc-373746, 1:200) were used. Secondary antibodies used were HRP-conjugated anti-rabbit IgG (Invitrogen, 31458, 1:1000) and anti-mouse (Invitrogen, SA1-100, 1:1000).

### 2.3 Transient depletion of NPC1 in hepatoma cell lines

HepG2-hNTCP, Huh7 and HepG2 2.2.15 cells seeded in either 24 or 48-well plates were treated with 50 nM NPC1 (Santa Cruz Biotechnology, sc-41588) or control siRNA (siCtrl) (Santa Cruz Biotechnology, sc-37007), for 48 (HepG2-hNTCP) or 72 hours (h) (Huh7 and HepG2 2.2.15), using lipofectamine RNAiMax (Invitrogen, 13778075) and the manufacturer’s protocol. NPC1 depletion was further confirmed by Western blotting.

### 2.4 Generation of the NPC1 knockdown HepaRG cell line

HepaRG cells with stable downregulation of NPC1 expression were obtained by using the commercially available CRISPR/Cas 9 system. Briefly, HepaRG cells were seeded in 6-well plates then co-transfected with 1 µg of each NPC1 CRISPR/Cas9 KO (Santa Cruz Biotechnology, sc-403252) and NPC1 HDR (Santa Cruz Biotechnology, sc-403252-HDR) plasmids, using lipofectamine 3000 (Invitrogen, L300015). Cell colonies selected in the presence of 1 µg/mL puromycin (Invivogen, ant-pr) were further expanded and denoted HepaRG NPC1-KD. Stable NPC1 knockdown was confirmed by Western blotting after each freeze-thaw cycle and multiple cell passages.

### 2.5 HBV production

Supernatants from HepG2 2.2.15 stably transfected with two copies of the HBV genome cell were collected every 3 days and clarified at 2320 g for 10 minutes. Viral particles were concentrated on a 20% sucrose cushion by ultracentrifugation at 32000 rpm (SW32Ti) for 5 h. The pellet was resuspended in DMEM containing 20% FBS, and the virus concentration was determined by real-time PCR, using serial dilutions of a pTriExHBV1.1. Concentrated virus was stored at -80 °C until further infection/internalization assays.

### 2.6 HBV infection

The 300-fold-concentrated HBV was used to infect HepG2-hNTCP at 300 genome equivalents (GEq)/cell and differentiated HepaRG cells at 1000 GEq/cell, following published protocols (Macovei et al., 2013; Pantazica et al., 2022). After 7 (HepG2-hNTCP) or 14 days (HepaRG), cells and media were harvested and HBV infection markers were analyzed. Cells treated with 1 µM Myrcludex B (also known as Bulevertide, Pepscan Therapeutics) for 3 h prior to viral inoculation were also included as control.

### 2.7 Pharmacological treatment design

Cells were treated with U18666A (MedchemExpress, 3039-71-2) or various FDA-approved drugs (TargetMoi, L4200), at concentrations indicated in figure captions, for 24 h prior to HBV infection. For time-of-addition type of experiments, HepG2-hNTCP cells were treated with 30 µM U18666A 48 h post HBV inoculation. To rule out a potential HBV-U18666A interaction, U18666A at 30 µM was incubated with 300 GEq/cell HBV 30 minutes at 37°C prior to HepG2-hNTCP viral infection.

### 2.8 Transfection of the HBV genome in Huh7 cells

Huh7 cells were transfected with pGEM-4Z-HBV 1.3WT (Addgene, 65459) for 24 h using lipofectamine 3000, then treated with 50 nM of either NPC1 or control (Ctrl) siRNA for another 72 h. At the end of experiment, cells and media were harvested and infection markers were assessed.

### 2.9 Exogenous EGFR expression in NPC1-inhibited HepG2-hNTCP cells

HepG2-hNTCP were transfected with 1 or 2 µg/mL of PWPXLD-EGFR-WT, encoding the wild-type EGFR in fusion with a HA tag (Addgene, 133749), for 6 h using lipofectamine 3000. Cells were then treated with 30 µM U18666A or DMSO for 24 h prior to HBV infection, as described above. EGFR expression was determined by Western blot at 24 h post-drug treatment. Cells treated with 1 µM Myrcludex B for 3 h before viral inoculation were also included as control. Secretion of HBeAg in cell supernatants was quantified by ELISA, at 7 days post-infection (dpi).

### 2.10 ELISA

Secretion of HBsAg and HBeAg from NPC1-depleted/inhibited cells was quantified by using the MonolisaHBsAg Ultra Kit (Bio-Rad, BIO72348) and Monolisa HBe Ag-Ab Kit (DiaPro, HBE.CE), respectively. The results are shown as percentage of HBsAg/HBeAg secretion relative to control.

### 2.11 Real-time PCR

Total or encapsidated HBV DNA was isolated from cell supernatants by phenol–chloroform extraction as described before (Lazar et al., 2007) and quantified by using the SensiFAST SYBR No-ROX kit (Bioline, BIO-86105) and a Corbett Rotor-Gene 6000 real-time PCR system (Qiagen). HBV GEq were calculated based on a standard curve generated from 10-fold serial dilutions of known concentrations of HBV DNA. The primers used for amplification were HBV_ forward (5′-TCCAGGATCCTCAACAACCAGCACG-3′) and HBV_reverse (5′-TGGCCCCCAATACCACATCATCC-3′). The total HBV DNA was normalized to the levels of the PRNP housekeeping gene using PRNP_forward 5’- TGCTGGGAAGTGCCATGAG-3’ and PRNP_reverse 5’- CGGTGCATGTTTTCACGATAGTA-3’ primers.

### 2.12 Reverse transcription real-time PCR

Total RNA was extracted from cells grown in 12-well plates by lysis in 1.0 mL of TRIzol (Life Technologies, Inc.), then subjected to RT and real-time PCR using the GoTaq one-step RT-qPCR kit (Promega, A6020), and the Corbett Rotor-Gene 6000 real-time PCR instrument. Specific primers used for amplification were forward: 5**’**-TGACCACCTGCTCCACCTC-3**’** (NTCP) and 5**’**-CGAGGGCAAATACAGCTT-3**’**(EGFR) and reverse: 5**’**-GAATGAGAACCAGGACCAGTGAT-3**’** (NTCP) and 5**’**-AAATTCACCAATACCTATT-3**’** (EGFR). Results were normalized using GAPDH as a reference gene by using primers forward: 5**’**-TGC ACC ACC AAC TGC TTA GC-3**’** and reverse 5**’**-ATG GCA TGG ACT GTG GTC TG-3**’**.

### 2.13 Immunoprecipitation of enveloped and non-enveloped HBV particles

Cell supernatants (500 µL) were incubated with either anti-core or anti-preS1 (antibodies, overnight at 4 °C. Next day, 15 µL of protein G–Sepharose beads (Life Technologies, 101242) were added for 3 h at 4 °C, to capture either nucleocapsids or enveloped HBV particles. The beads were washed five times with phosphate-buffered saline (PBS), and bound virions were eluted using 50 mM Tris–HCl buffer (pH 8) (Milipore, 9310) containing 1 mM EDTA (Sigma, E6635) and 1% Nonidet P-40 (Sigma, I8896). The viral DNA was subsequently isolated and quantified as described above.

### 2.14 MTS assay

The CellTiter 96^®^ AQueous One Solution Cell Proliferation Assay (Promega, G3580), was performed to determine the cytotoxicity of tested molecules on indicated cells. In brief, cells were exposed to U18666A, itraconazole, posaconazole, verapamil or amiodarone at concentrations indicated in the figure legends. After incubation for 24 h, TS tetrazolium (Owen’s reagent) was added to the medium and the ODs at 490 nm were recorded after 1.5 h.

### 2.15 Western blotting

Cells were lysed on ice for 30 minutes in RIPA buffer containing 50 mM Tris-HCl (pH 7.4), 150 mM NaCl, 1 mM EDTA, 1% Triton X-100 (Sigma, T8787) 1% sodium deoxycholate, and 0.1% SDS, supplemented with 1x protease inhibitor cocktail (Santa Cruz Biotechnologies) and 1mM sodium orthovanadate, 1mM NaF, 50mM β-glycerophosphate, and 10 mM N-ethylmaleimide. Where indicated, cell lysates were treated with Endo H (New England Biolabs, P0702S) or PNGase F (New England Biolabs, P0704S), following the producer’s protocol. Equal amounts of total cell lysates protein along with protein ladders (Termo Fisher Scientific, 26616 and 26623) were separated by sodium dodecyl sulfate-polyacrylamide gel electrophoresis (SDS-PAGE) and transferred onto polyvinylidene difluoride (PVDF; Santa Cruz Biotechnology, SC-3723) membranes using a wet-tank blotting system (Bio-Rad). Membranes were blocked with 5% bovine serum albumin (BSA; Sigma-Aldrich, A9647-100G) and incubated overnight at 4 °C with primary antibodies, followed by secondary antibodies for 1 h at room temperature. Membranes were incubated with enhanced chemiluminescence (ECL; Thermo Fisher Scientific, 32209) or SuperSignalTM West Femto substrate (Thermo Fisher Scientific, 34096), followed by protein detection on autoradiography films or by using the ChemiDoc MP system (BioRad). Proteins of interest were quantified by using the ImageJ software (National Institutes of Health).

### 2.16 Immunofluorescence microscopy

Cells were seeded onto collagen-coated coverslips (19 mm diameter) and allowed to adhere and then 30 µM U18666A or DMSO were added for 24 h. Subsequently, cells were fixed with 1% paraformaldehyde (PFA) for 5 minutes at room temperature and permeabilized with 0.1% Triton X-100 (Sigma-Aldrich, T8787-250ML). For NTCP staining, fixation was performed with 4% paraformaldehyde (PFA; Sigma-Aldrich, P6148-500G) for 20 minutes. Following fixation, samples were blocked with 1% BSA in PBS, for 1 h at room temperature and then incubated with the appropriate primary and secondary antibodies for 30 minutes at room temperature. For filipin staining, HepG2-hNTCP cells seeded on coverslips were treated with either 30 µM U18666A or DMSO for 24 h. Cells were then washed with PBS, fixed with 2% PFA for 10 minutes, and washed again with PBS. Following fixation, cells were quenched with 50 mM glycine for 10 minutes and incubated with 100 µg/mL filipin III (Sigma, F4767) for 2 h. Nuclei were stained with 4’6-diamidino-2-phenylindole (DAPI; Thermo Fischer Scientific, 62248). Images were acquired using a Zeiss LSM 730 confocal laser scanning microscope (Carl Zeiss Microscopy) equipped with a 63x oil-immersion objective. Overlapping fluorescence was quantified by measuring the Mander’s M1 co-localization coefficient. Image processing and analysis were performed using ImageJ software (National Institutes of Health).

### 2.17 In-cell ELISA

NPC1-depleted/-inhibited HepG2-hNTCP cells grown in 96-well plates were fixed with either 1 or 2% PFA for EGFR and NTCP detection, respectively, for 5 minutes at room temperature. Excess PFA was quenched with 50 mM glycine (Merck, 100590). Cells were washed with PBS, blocked with 5% non-fat milk for 45 minutes and incubated with primary antibodies against NTCP or EGFR for 30 minutes. Samples were incubated with HRP-conjugated secondary antibodies for 30 minutes, before addition of the TMB (BD OptEiA, 555214) substrate. The colorimetric signal was measured at 450 nm after 30 minutes, using the Mithras LB940 Microplate Reader (Berthold Technologies). All incubations were performed at room temperature.

### 2.18 Cycloheximide-chase assay

To monitor the degradation kinetics of EGFR, HepG2-hNTCP cells were seeded and pre-treated for 24 h with 30 µM U18666A or DMSO as control. Subsequently, the culture medium was replaced with fresh medium supplemented with 50 µM cycloheximide (CHX; Sigma-Aldrich, 239763) to inhibit de novo protein synthesis, maintaining U18666A treatment where applicable. Cells were harvested at the indicated time points for kinetic analysis. To determine the specific proteolytic pathway responsible for EGFR clearance, cells pre-treated with U18666A for 24 h were incubated with fresh medium containing CHX, U18666A, and either the lysosomal cysteine protease inhibitor E-64 (20 µM; Santa Cruz Biotechnology, sc-201280) or MG132 (25 µM; Santa Cruz Biotechnology, sc-201036), to avert EGFR ubiquitination. Cells were then incubated for an additional 3 h prior to harvesting. Following treatments, cells were lysed, and equal amounts of total protein were resolved by SDS-PAGE and transferred onto PVDV membranes. EGFR levels were analyzed via Western blotting using specific antibodies. Densitometric analysis of immunoreactive bands was performed using ImageJ software. EGFR levels were normalized to their respective calnexin loading controls. The degradation half-life (t1/2) of EGFR was calculated by fitting the normalized values to a one-phase exponential decay model using GraphPad Prism software.

### 2.19 HBV internalization

HepG2-hNTCP cells were seeded in 24-wells collagen-coated plates, and the following day were treated with either U18666A (30 μM) or DMSO, as control. At 24 h post-treatment, cells were inoculated with 300 GEq/cell HBV for 2 h at 4 °C then washed five times with media and five times with PBS to remove unbound virions. After addition of fresh medium, cells were incubated at 37 °C, for 16 h to allow for virus internalization. After another five washes with PBS, cells were treated with trypsin before centrifugation at 10000 g, for 10 minutes then lysed with buffer containing 50 mM Tris-HCl (pH 8), 1 mM EDTA and 1% Nonidet P-40. Total DNA was extracted as described (Lazar et al., 2007), except for the RNase and DNase treatment. HBV DNA was measured by real-time PCR and normalized to PRNP gene levels (Dobrica et al., 2024). As control, Myrcludex B (1 μM) was added to cells 3 h prior viral inoculation and was maintained throughout the assay.

### 2.20 Statistical analysis

Graphs were generated using GraphPad Prism for Windows (version 8; GraphPad Software, San Diego, CA, USA). Data are shown as mean ± SD from two or three independent experiments. Statistical significance was determined as indicated in figure legends. Statistical significance was considered at p 0.05 for all analyses.

## 3 Results

### 3.1 NPC1 depletion and chemical inhibition result in significant inhibition of HBV infection

**Figure 1:**
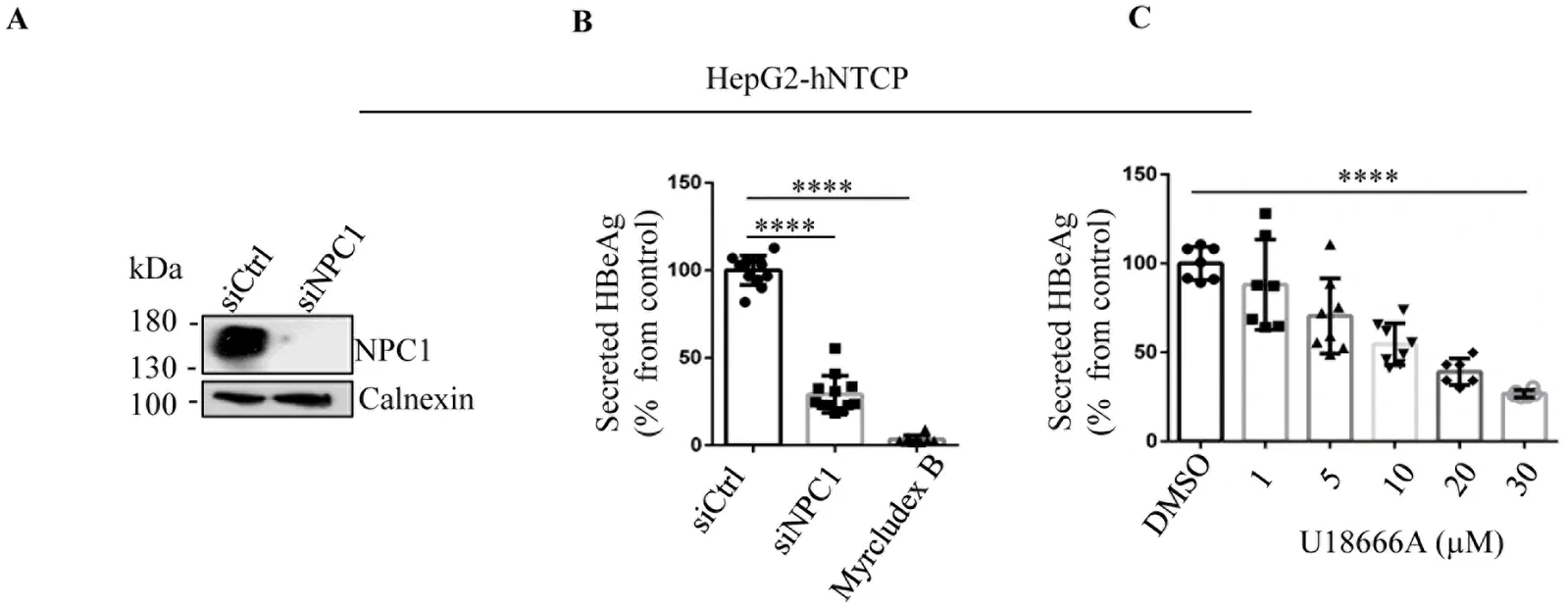
NPC1 depletion and chemical inhibition result in significant decrease of HBV infection in HepG2-hNTCP cells. **(A)** HepG2-hNTCP cells were transfected with 50 nM of either NPC1 or control (Ctrl) siRNA. NPC1 expression was detected by Western blot at 48 h post-transfection. Calnexin detection was used as protein loading control. One representative experiment is shown (n = 4). **(B, C)** HepG2-hNTCP cells were treated with either siRNA for 48 h, 1 μM Myrcludex B for 3 h or U18666A at indicated concentrations for 24 h, before infection with 300 GEq/cell HBV. Secreted HBeAg was quantified by ELISA at 7 dpi. Data are shown as mean ± SD from four **(B)** or three **(C)** independent experiments performed in three **(B)** or two **(C)** biological replicates. Statistical significance was determined using a two-tailed Student’s t-test **(B)** or one-way ANOVA **(C)** (****p < 0.0001).

To evaluate the role of NPC1 in HBV infection, HepG2-hNTCP cells were treated with NPC1 siRNA or a scrambled siRNA sequence, as control. Analysis of NPC1 expression by Western blotting indicated efficient downregulation of the target protein at 48 h post-transfection (**Figure 1A**). HBV infection in siRNA-treated cells was further monitored by quantification of the HBeAg in cell supernatants at 7 dpi. Myrcludex B, an efficient inhibitor of HBV entry was also included in the experiment to control for the specificity of infection (Urban et al., 2014). The analysis revealed a strong inhibition of HBV infection in cells with reduced NPC1 expression suggesting a role of this protein in the viral life-cycle (**Figure 1B**). To confirm this hypothesis, we used the pharmacological NPC1 inhibitor U18666A, which binds to the sterol-sensing domain of the protein, preventing the transport of cholesterol out of the lysosomes (Lu et al., 2015). A dose-dependent inhibition of the HBeAg secretion from infected cells was observed in the presence of U18666A (**Figure 1c**), at concentrations that maintained cell viability, as determined by MTS assays (**Supplementary Figure 1**). Filipin staining following treatment with U18666A revealed the intracellular accumulation of unesterified cholesterol in a punctate-like pattern expected for the NPC1 phenotype (Kwon et al., 2009), confirming the inhibitory effect on cholesterol transport in HepG2-hNTCP cells (**Supplementary Figure 2**).

**Figure 2:**
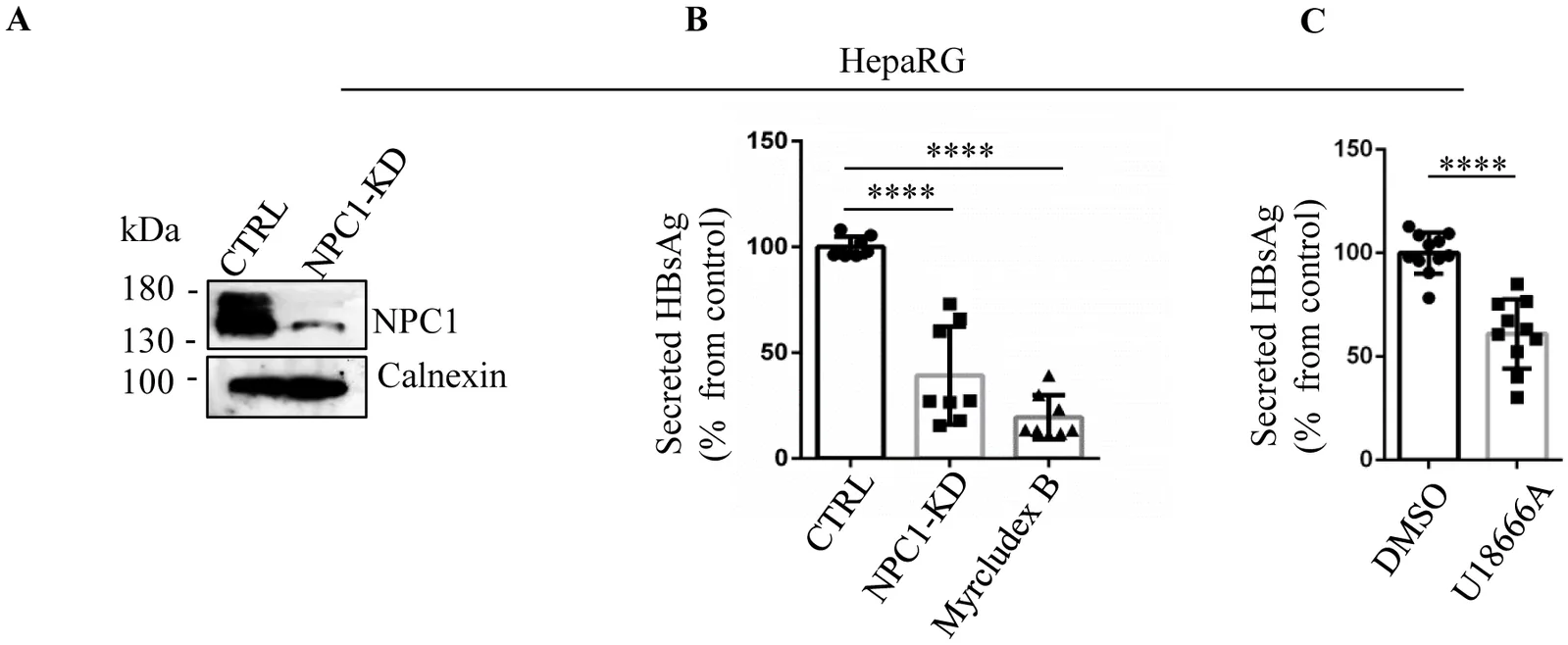
HBV infection is inhibited in NPC1 knockdown (KD) HepaRG cells. **(A)** NPC1 expression in wild-type (CTRL) and NPC1-KD HepaRG cells was determined by Western blot. Calnexin was used as protein loading control. One representative experiment (n = 4) is shown. **(B, C)** HepaRG cells were differentiated for 4 weeks prior to infection with 1000 GEq/cell HBV. Where indicated, HepaRG CTRL cells were treated with 30 µM U18666A or DMSO for 24 h before infection. Treatment with 1 μM Myrcludex B was included as control for inhibition of infection. Secreted HBsAg was quantified by ELISA at 14 dpi. Data are shown as mean ± SD from three independent experiments performed in three biological replicates. Statistical significance was determined using a two-tailed Student’s t-test (****p < 0.0001).

To validate the experimental observations described above, we next used HepaRG cells, a hepatic progenitor cell line with a close phenotype to primary human hepatocyte (PHH) that are considered as the best surrogate model to investigate PHH-related functions *in vitro*. Differentiated (d) HepaRG cells are permissive to HBV infection, and unlike other hepatoma- derived cell lines, express key molecules of the innate immune system that are relevant in the context of viral-host proteins interactions (Gripon et al., 2002). As transient transfection is rather inefficient in dHepaRG, CRISPR/Cas9 gene-edited NPC1 knockdown HepaRG cells (NPC1-KD) were generated and then differentiated, as previously described (Gripon et al., 2002). Western blot analysis confirmed a strong downregulation of NPC1 in these cells, as compared to control (**Figure 2A**). Moreover, the HBV infection was significantly inhibited in both NPC1-KD (**Figure 2B**) and wild-type HepaRG cells treated with U18666A (**Figure 2C**), confirming a pro-viral role for NPC1 in HBV infection.

### 3.2 NPC1 depletion modulates HBV entry with no impact on late stages of HBV infection

**Figure 3:**
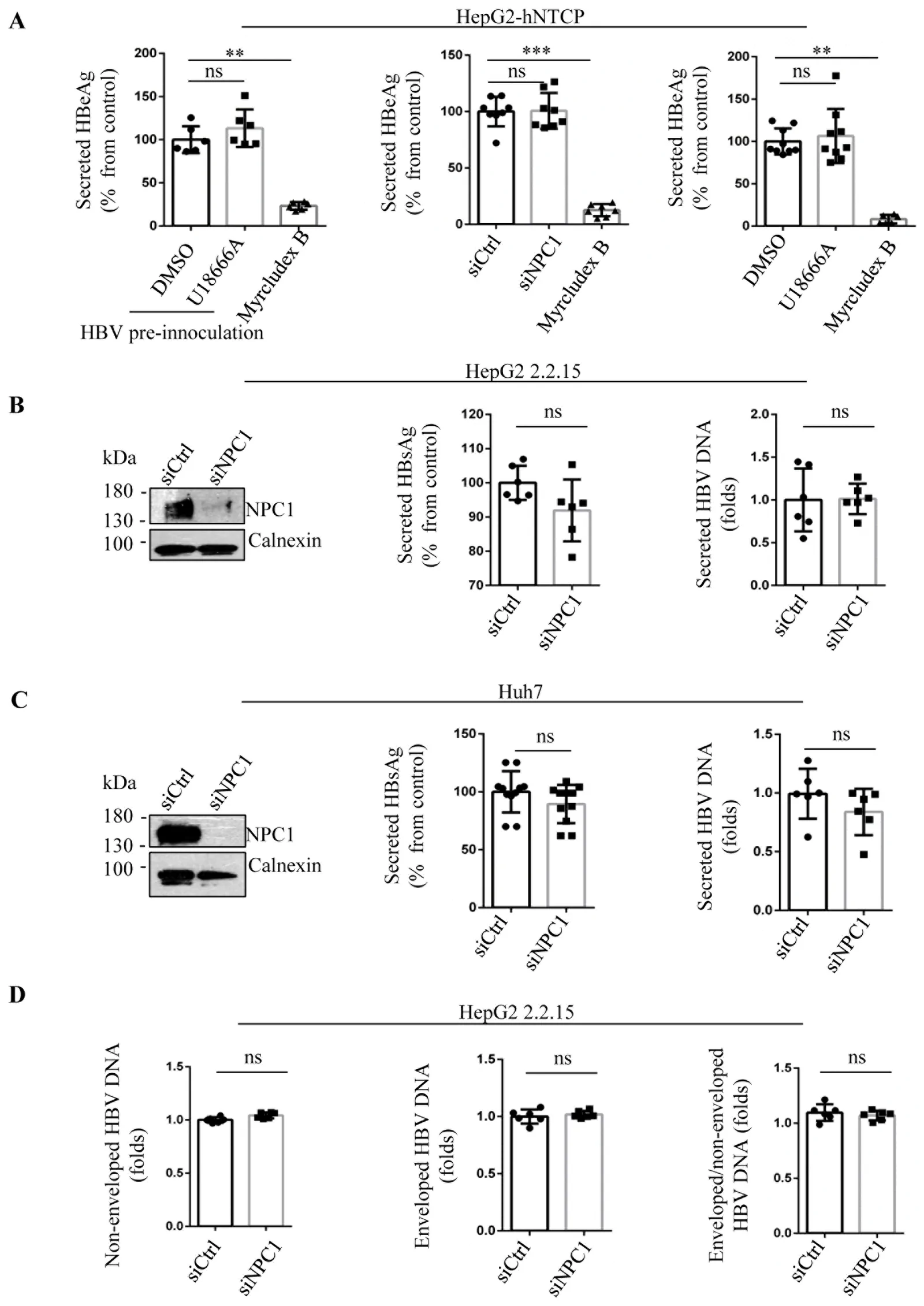
NPC1 depletion modulates HBV internalization with no impact on late stages of HBV infection. **(A)** HBV inoculum (300 GEq/cell) was incubated with 30 µM U18666A or DMSO for 30 min prior to inoculation of HepG2-hNTCP (left panel). HepG2-hNTCP cell were infected with 300 HBV GEq/cell then treated with either siRNA (middle panel) or 30 µM U18666A (right panel) at one and 2 dpi, respectively. Cells pre-treated with 1 μM Myrcludex B for 3 h were included as control for inhibition of infection. Secreted HBeAg was quantified by ELISA at 7 dpi. **(B)** HepG2.2.15 cells were transfected with 50 nM of either NPC1 or control (Ctrl) siRNA. NPC1 expression was detected by Western blot at 48 h post-transfection. Calnexin detection was used as protein loading control. One representative experiment is shown (n = 2, left panel). Secreted HBsAg (middle panel) and viral nucleocapsids (right panel) were quantified by ELISA and qPCR, respectively. **(C)** Huh7 cells were transfected with pGEM-4Z-HBV 1.3WT then treated with 50 nM of either NPC1 or control (Ctrl) siRNA. NPC1 expression was detected by Western blot at 72 h post-transfection. Calnexin detection was used as protein loading control. One representative experiment is shown (n = 2, left panel). Secreted HBsAg (middle panel) and viral nucleocapsids (right panel) were quantified by ELISA and qPCR, respectively. **(D)** HepG2.2.2.15 cells were transfected with 50 nM of either NPC1 or control (Ctrl) siRNA for 72 h. Cell supernatants were subjected to immunoprecipitation with anti-core (left panel) or anti-preS1 antibodies (middle panel) to pull down non-enveloped and enveloped viral particles, respectively, followed by HBV DNA extraction and quantification by real-time PCR. The ratio between enveloped and non-enveloped HBV is also shown (right panel). All data are presented as mean ± SD from at least two independent experiments performed in 3 biological replicates. Statistical significance was determined using a two-tailed Student’s t-test (t-test (***p < 0.001, **p<0.01, ns, not significant).

In the experiments described above, NPC1 silencing and pharmacological inhibition preceded HBV inhibition, therefore both early and late steps of the HBV life-cycle could be affected by the treatment. Because of the U18666A structure as an amphipathic cationic amine, its direct interaction with the viral envelope or the surface proteins affecting HBV infectivity is also possible. To discriminate between these scenarios, the HBV inoculum was pre-treated with the highest U18666A concentration that prevented inhibition of HBV infection, before addition to HepG2-hNTCP cells. As shown in **Figure 3A** (left panel), this treatment had no consequences on HBV infection, indicating that U18666A does not inactivate the virus. Similarly, NPC1 silencing (**Figure 3A**, middle panel) or chemical inhibition (**Figure 3A**, right panel) occurring post-HBV inoculation, did not affect infection, suggesting that viral entry, rather than replication is impaired by the treatment.

To explore this hypothesis, HBV replication was further investigated in two complementary cell systems that are not permissive for HBV infection, HepG2 2.2.15 cells containing two copies of the HBV genome (**Figure 3B**) and Huh7 cells transiently transfected with a replication-competent HBV plasmid (**Figure 3C**). A strong downregulation of NPC1 expression was first confirmed in cells treated with the corresponding siRNA, as compared with controls (**Figures 3B, C**, left panels). Interestingly, the levels of HBsAg (**Figures 3B, C**, middle panels) and viral particles (**Figures 3B, C**, right panels) in cell supernatants were not affected by NPC1 depletion, indicating no significant impact on HBV replication and extracellular release.

The HBV DNA quantified in cell supernatants originates from fully enveloped virions and/or nucleocapsids. Since cell depletion of cholesterol may alter the transmembrane topology of the HBV-L envelope protein and impair HBV envelopment (Dorobantu et al., 2011), we next addressed the possibility that NPC1 downregulation would produce a similar effect on HBV assembly. Cell supernatants from NPC1-silenced and control HepG2 2.2.15 cells were subjected to immunoprecipitation with either anti-preS1 (to isolate the enveloped virions) or anti-core antibodies (to pull down non-enveloped HBV nucleocapsids). Quantification of the HBV DNA purified from bound immune complexes indicated similar amounts of enveloped and non-enveloped viral particles (**Figure 3D**), suggesting no role for NPC1 on HBV morphogenesis. Collectively, these results support the conclusion that NPC1 is not involved in the late steps of HBV infection.

### 3.3 NPC1 depletion and pharmacological inhibition results in decreased expression of the HBV co-receptor, EGFR and impaired viral particles internalization

**Figure 4:**
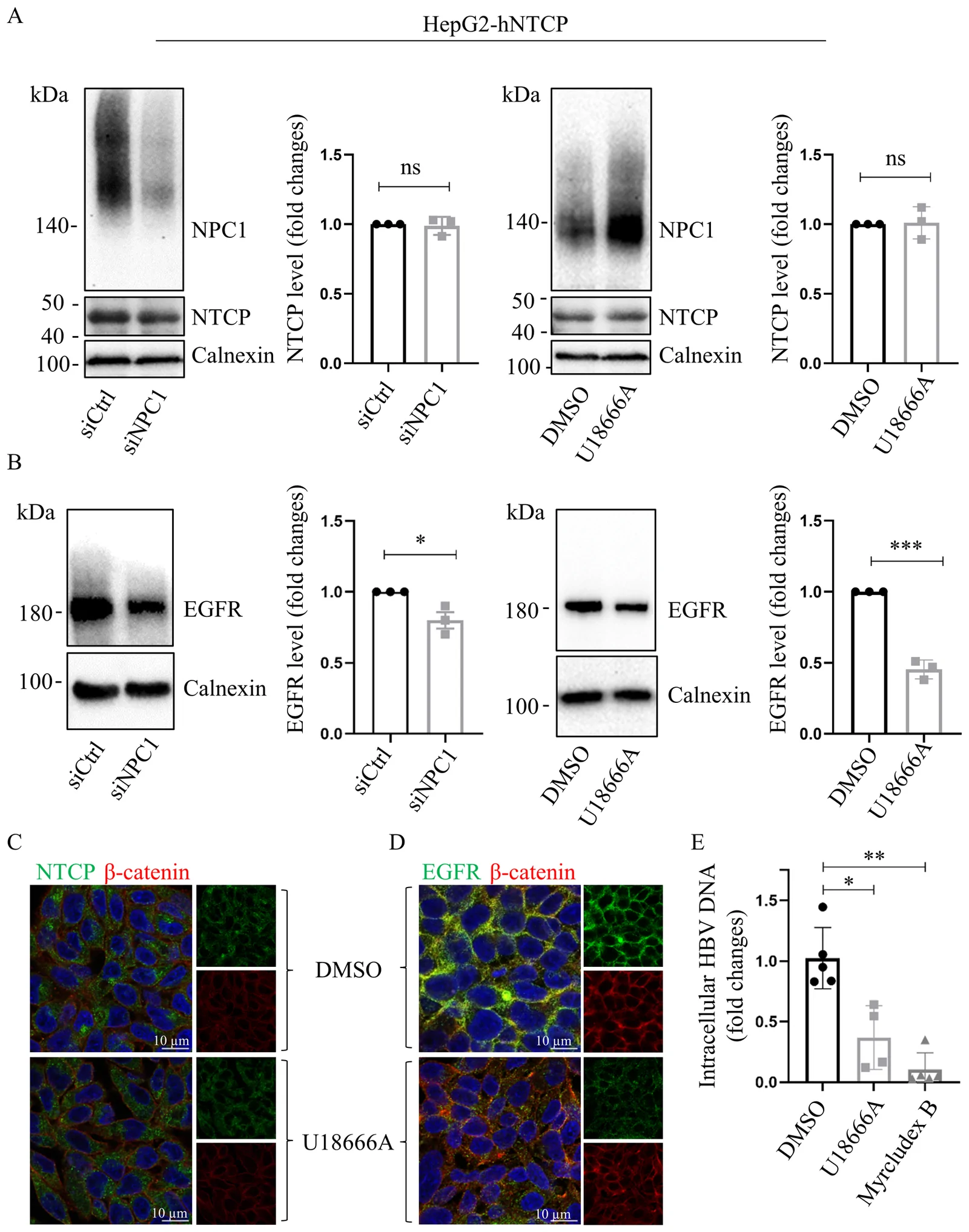
NPC1 inhibition reduces EGFR expression and HBV internalization in HepG2-hNTCP cells. **(A)** NTCP protein expression was analyzed in HepG2-hNTCP cells transfected with NPC1-targeting siRNA (siNPC1) or non-targeting control siRNA (siControl) for 48 h (left panel) or treated with 30µM U18666A or vehicle control (DMSO) for 24 h (right panel). **(B)** EGFR protein expression levels were analyzed in HepG2-hNTCP cells treated as described in A). Whole-cell lysates were prepared, and equal amounts of total protein were analyzed by Western blot using antibodies against NTCP, EGFR and NPC1. Calnexin was used as a loading control. Densitometry analysis was performed, and results are presented as fold change relative to control. Data are shown as mean ± SD from three independent experiments. Statistical significance was determined using an unpaired two-tailed Student’s t-test (ns, not significant; *p < 0.05; ***p < 0.0001). **(C, D)** For subcellular localization studies, HepG2-hNTCP cells were treated with 30 µM U18666A or DMSO for 24 h. Representative immunofluorescence images show NTCP (green) and β-catenin (red) staining **(C)**, and EGFR (green) and β-catenin (red) staining **(D)**. Nuclei were counterstained with DAPI. Scale bar: 10 µm. **(E)** HepG2-hNTCP cells were treated with 30 μM U18666A or DMSO for 24 h prior to HBV inoculation at 300 GEq/cell, for 2 h at 4 °C. After 16 h, total HBV DNA was extracted from cells, quantified by real-time PCR and normalized to PRNP gene levels. Cells treated with 1 μM Myrcludex B served as control. Data are presented as fold changes from DMSO control levels. The means ± SD from two independent experiments performed in three biological replicates are shown. Statistical analysis was performed using Mann-Whitney U test (*p < 0.05, **p < 0.01).

The data described above point to NPC1 inhibition/depletion interfering at an early step of HBV infection. This observation prompted us to investigate the expression of the HBV receptors, NTCP and EGFR under the same experimental conditions. As shown in **Figure 4A**, neither NPC1 depletion nor the U18666A treatment affected the NTCP levels, as detected by Western blotting. In contrast, significantly lower amounts of EGFR were detected in NPC1 siRNA- or drug-treated HepG2-hNTCP cells, by 30 and 50% respectively, as compared with corresponding controls (**Figure 4B**). As a direct inhibitor of cholesterol binding to NPC1, it is likely that U18666A may perturb more efficiently the lysosomal cholesterol efflux, when compared to genetic NPC1 depletion, and thus induce the stronger EGFR phenotype observed in drug-treated cells (**Figure 4B**).

Further support of the contrasting behavior between the two HBV receptors was provided by a more quantitative evaluation of NTCP and EGFR expression, using In-Cell ELISA (**Supplementary Figure 3**). The intracellular distribution of NTCP and EGFR was further investigated in more depth by fluorescence microscopy. Since U18666A successfully mimicked the loss of functional NPC1 and had a more pronounced effect on EGFR, all immunofluorescence assays were performed in cells treated with this drug. Expression of β-catenin, a key component of cell adhesion junctions, was also determined to specifically mark the plasma membrane (Liu et al., 2022). NTCP exhibited a similar fluorescence pattern, with both plasma membrane and intracellular localization, regardless the drug treatment (**Figure 4C**). By contrast, U18666A-treated cells exhibited a clear decrease in EGFR abundance at the plasma membrane, concurrent with the localization of the protein in cytoplasmic bright puncta (**Figure 4D**). This observation confirms that NPC1 inhibition causes an overall depletion of cellular EGFR and suggests a re-location of the receptor from the plasma membrane to intracellular compartments.

Since EGFR facilitates HBV endocytosis and routing of viral particles along the endocytic pathway (Iwamoto et al., 2019) we next investigated whether depletion of the receptor from the plasma membrane had any impact on HBV uptake. Indeed, as shown in **Figure 4E**, a lower level of viral DNA was detected in U18666A-treated HepG2-hNTCP cells at 16 h post-virus inoculation, suggestive of impaired HBV internalization.

### 3.4 Significant EGFR degradation occurs in NPC1-depleted/pharmacologically-inhibited cells

**Figure 5:**
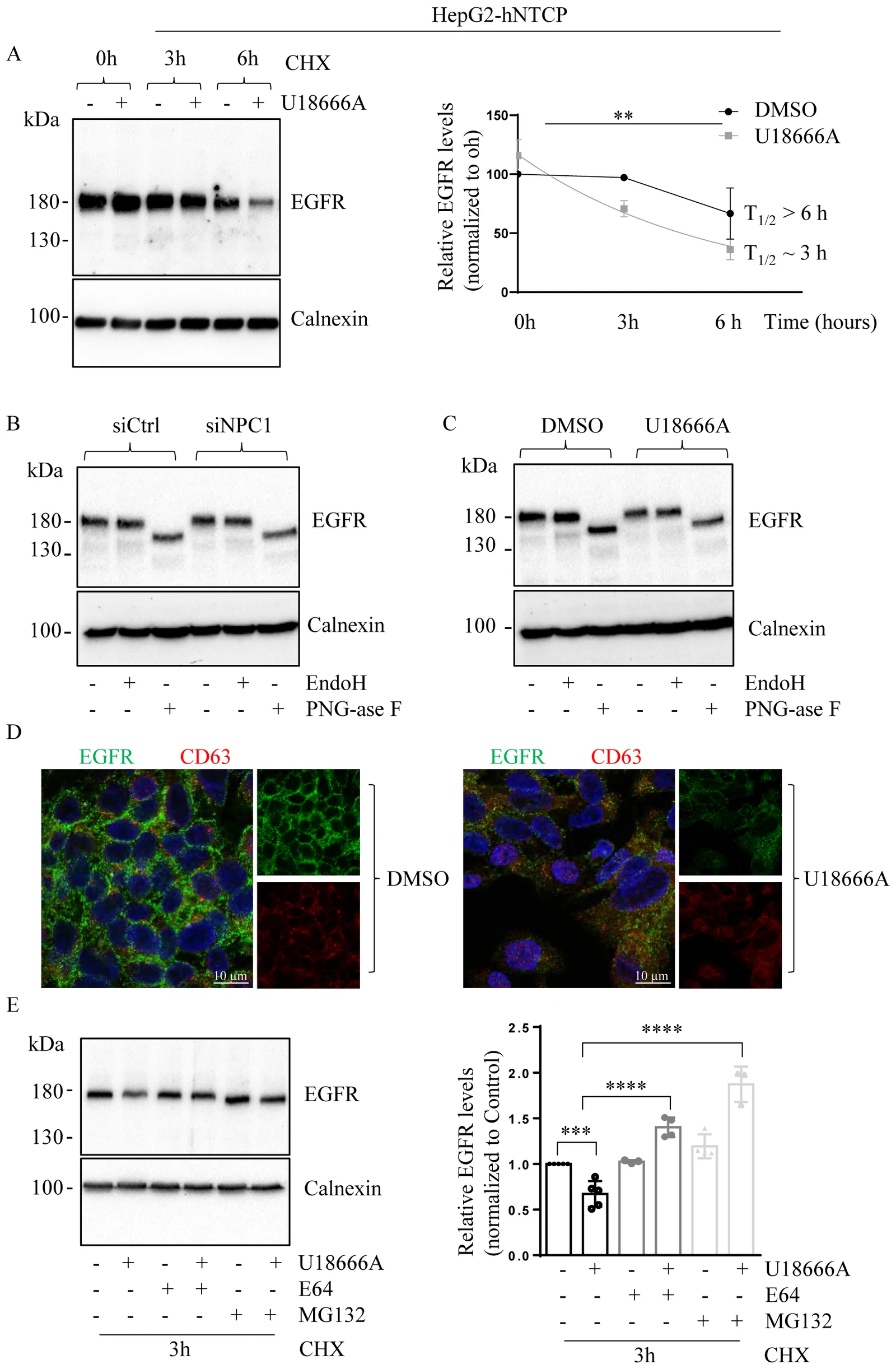
NPC1 deficiency/inhibition promotes EGFR degradation. **(A)** EGFR degradation kinetics was evaluated using CHX- chase assays. HepG2-hNTCP cells were treated with 30 µM U18666A for 24 h and subsequently incubated with fresh medium containing 50 µM CHX in the presence or absence of 30 µM U18666A. Cells were harvested at the indicated time points and EGFR levels were analyzed by Western blot. EGFR half-life (t1/2) was determined by densitometry analysis. Data were fitted using a one-phase decay non-linear regression model with plateau constrained to 0 in GraphPad Prism. Differences between degradation curves were assessed using the extra sum-of-squares F-test. Data are presented as mean ± SD from two independent experiments; **p < 0.01. **(B-C)** HepG2-hNTCP cells were either transfected with siRNA targeting NPC1 (siNPC1) or a random control siRNA sequence (siCtrl) for 48 h **(B)** or treated with 30µM U18666A or DMSO for 24 h **(C)**. Cell lysates were digested with Endo H or PNGase F and analyzed by Western blot using anti-EGFR antibodies. **(D)** For immunofluorescence analysis, cells were treated with 30 µM U18666A or DMSO for 24 h and stained with anti-EGFR or anti-CD63 (Lamp3) antibodies, followed by Alexa Fluor 488-conjugated secondary antibodies. **(E)** CHX-chase experiments were performed as described in **(A)**. Cells were treated with 10 µM U18666A for 24 h, followed by incubation with 20 µM E-64 or 25 µM MG132 for 3 h prior to harvesting. EGFR levels were analyzed by Western blot and quantified by densitometry analysis relative to untreated controls. Statistical analysis was performed using a two-tailed Student’s t-test (***p < 0.001, ****p < 0.0001).

The loss of EGFR expression in NPC1- depleted/inhibited cells can be accounted for by an impaired mRNA transcription or protein degradation. Quantification of NTCP and EGFR transcripts revealed similar amounts in U18666A-treated and control HepG2-hNTCP cells, indicating a regulation of protein expression at post-transcriptional level (**Supplementary Figure 4**). To further investigate the mechanism underlying EGFR stability in NPC1-inhibited cells, a CHX-chase assay was performed, to stop protein translation and evaluate the degradation kinetics of the receptor more accurately. EGFR expression in the presence and absence of U18666A was monitored for up to 6 h after addition of CHX. A significant reduction of the EGFR half-life was observed in drug-treated (~ 3 h) as compared with control cells (> 6 h), indicating a role for the cholesterol transport function of NPC1 in EGFR homeostasis (**Figure 5A**).

EGFR degradation is a complex and tightly regulated process involving two main pathways. Newly synthesized EGFR may trigger endoplasmic reticulum (ER) stress leading to proteasomal disposal via the Unfolded Protein Response (UPR) and the ER-associated degradation, which reduces the protein pool reaching the plasma membrane (Miura et al., 2023; Shen et al., 2023). Plasma membrane-localized EGFR is internalized by clathrin-dependent or independent endocytosis, following activation by EGF and ubiquitination (Mosesson et al., 2003; Huang et al., 2006; Orth et al., 2006; Soldati and Schliwa, 2006), a process inhibited by proteasome inhibitors, such as bortezomib and MG132 (Kesarwala et al., 2009). Ubiquitination is a key modulator of the EGFR fate, routing the receptor through the endosomal pathway to the late endosomes for further degradation in lysosomes. In contrast, EGFR de- ubiquitination, by specific enzymes, prevents its transport from early endosomes to the lysosomes and promotes recycling back to the cell surface (Eden et al., 2012).

To gain mechanistic insights into the EGFR trafficking in NPC1-depleted/inhibited cells, we took advantage of the protein being highly glycosylated, mostly with complex-type N-linked oligosaccharides, characteristic of specific processing in the trans-Golgi network (Zhen et al., 2003). Cell lysates were treated with Endo H, an enzyme which removes high mannose oligosaccharides from N-linked glycoproteins, while complex glycans are resistant to treatment. Protein digestion with PNGase F, a glycoamidase which removes N-glycans from glycoproteins regardless of their processing, was also included as control. PNGase F treatment resulted in increased electrophoretic mobility of EGFR in both control and NPC1-depleted/inhibited samples, corresponding to the expected reduction of the molecular size due to the loss of the N-linked glycans. In contrast, the protein was entirely insensitive to Endo H digestion under the same experimental condition (**Figures 5B, C**). Quantification of the EGFR levels by densitometry analysis, confirmed the instability of the receptor in NPC1-depleted/inhibited cells (**Supplementary Figure 5**). Together, the data indicate that the loss of NPC1 or its function does not prevent EGFR from moving along the secretory pathway to the plasma membrane and therefore, the observed protein degradation is likely a post-ER event. This hypothesis is supported by the immunofluorescence microscopy analysis, showing an increased localization of EGFR in CD63 (Lamp3)-positive compartments in the presence of U18666A (**Figure 5D**), as quantified by the Mander’s M1 co-localization coefficient (**Supplementary Figure 6**), suggestive of enhanced routing of the receptor towards the late endocytic pathway. Importantly, cell treatment with either MG132, to avert EGFR ubiquitination (Kesarwala et al., 2009) or the thiol proteinase inhibitor E-64 that acts selectively on lysosomal protein degradation (Grinde, 1982), efficiently reversed the EGFR degradation induced by U18666A (**Figure 5E**). Together, the data reinforce the concept that NPC1 loss of function promotes EGFR trafficking from the plasma membrane to lysosomes where the receptor is degraded.

Importantly, the HA-tagged EGFR, exogenously expressed in an attempt to investigate a potential rescue of HBV infection in NPC1-inhibited cells, was also significantly degraded in the presence of U18666A (**Supplementary Figures 7A, B**). The PNGase F treatment of cell lysates validated the presence of N-linked glycans attached to both endogenous and overexpressed EGFR (**Supplementary Figure 7C**). Quantification of HBeAg in supernatants of drug-treated cells indicated a slight recovery of HBV infection for the highest concentration of plasmid encoding the HA-tagged EGFR used for cell transfection (**Supplementary Figure 7D**). The implication of these findings is further analyzed in Discussion.

### 3.5 HBV infection is restricted by FDA-approved drugs that inhibit NPC1 function or induce a NPC1-like phenotype

It has been previously shown that a number of FDA-approved small molecules including the antifungal drugs itraconazole and posaconazole, inhibit lysosomal cholesterol export by direct binding to NPC1 (Trinh et al., 2017). Similarly, amiodarone, a multi-ion channel inhibitor and adrenoceptor antagonist and the L-type calcium channel blocker verapamil, inhibit cholesterol egress from lysosomes, inducing a NPC1-like phenotype in treated cells (Piccoli et al., 2011; Balmith et al., 2017).

Interestingly, repurposing of these families of drugs has proved efficient in inhibiting Influenza, Ebola or Semliki Forest virus infections, by regulating cholesterol homeostasis (Gehring et al., 2014; Schloer et al., 2019; Varghese et al., 2022), suggesting a potential broad antiviral effect.

**Figure 6:**
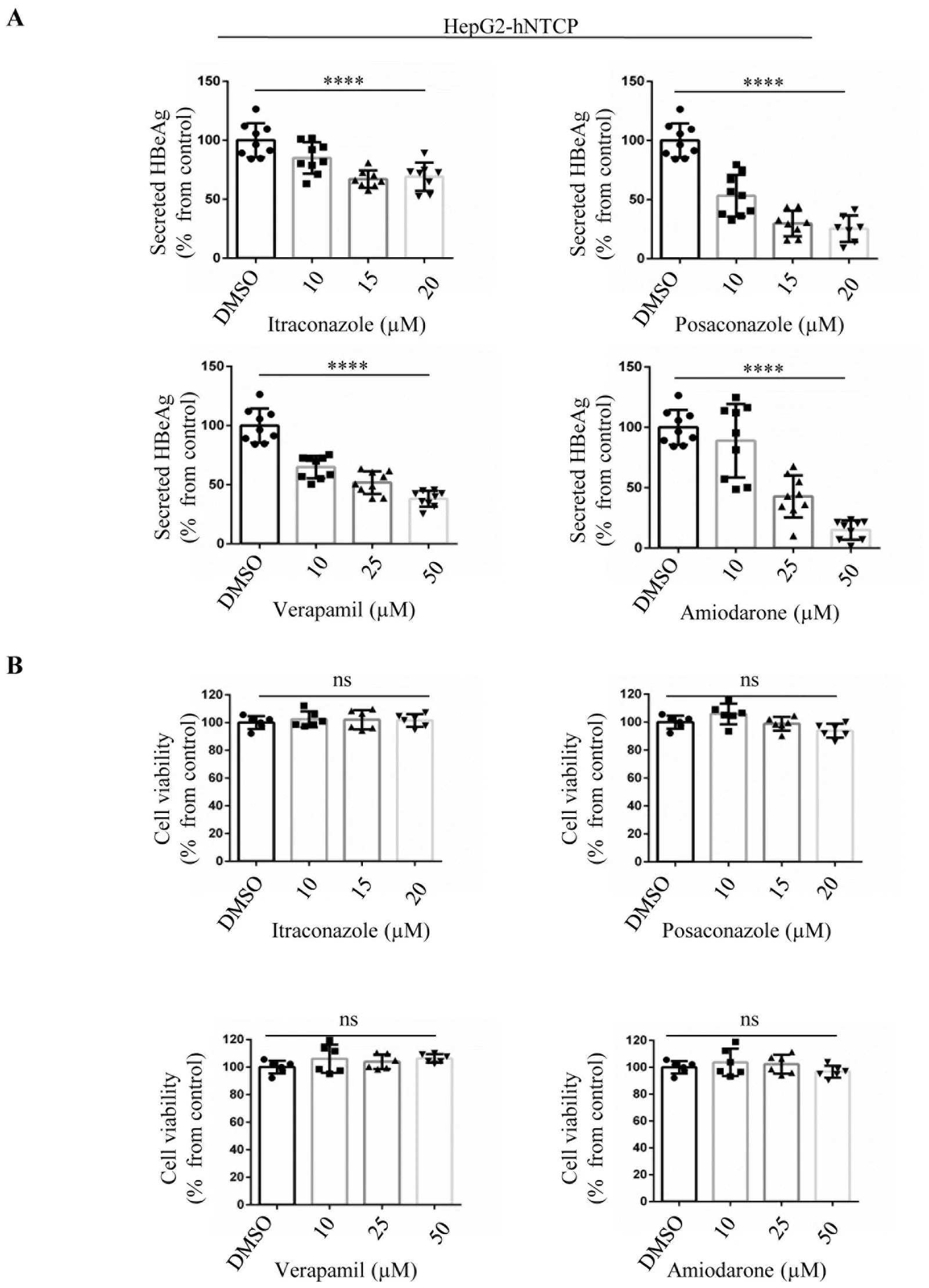
FDA-approved NPC1 inhibitors reduce HBV entry. **(A)** HepG2-hNTCP cells were treated with either itraconazole, posaconazole, verapamil, amiodarone at the indicated concentrations, or DMSO for 24 h prior to HBV inoculation (300 GEq/cell). Secreted HBeAg levels were quantified by ELISA at 7 dpi. **(B)** Drug toxicity was evaluated by MTS assay, after 24 h of incubation. Data are presented as mean ± SD percentages from DMSO controls from three **(A)** or two **(B)** independent experiments performed in at least two biological replicates. Statistical significance was determined by using one-way ANOVA (****p < 0.0001, ns, not significant).

Considering the NPC1 role in HBV entry established in this study, we next aimed to evaluate a potential inhibition of HBV infection by these drugs. HepG2-hNTCP cells were treated with itraconazole, posaconazole, verapamil or amiodarone at the indicated concentrations, before HBV inoculation. Interestingly all compounds showed significant inhibitory activity against HBV infection (up to 75%), as measured by quantification of the HBeAg in cell media (**Figure 6A**) at concentrations that did not compromise cellular viability (**Figure 6B**). The obtained results promote repurposing of these families of clinically approved drugs as an encouraging strategy for discovery of novel HBV inhibitors.

## 4 Discussion

Due to the crucial role in the lysosomal cholesterol transport, NPC1 regulates the cellular sterol homeostasis with direct impact on the lipid bilayer fluidity, transmembrane protein localization and receptor signaling at the plasma membrane and intracellular organelles. It is therefore not surprising that the dynamics and maturation of organelles in the endocytic pathway and trafficking of cargoes are significantly altered in NPC1-deficient cells (Lloyd-Evans et al., 2008; Rus et al., 2023).

The EGFR- cellular cholesterol homeostasis relationship is particularly interesting and complex. It has been shown that normal cholesterol levels restrict EGFR diffusion at the plasma membrane by preventing spontaneous activation, dimerization and internalization in the absence of ligand binding (Takayama et al., 2024). More recently, a direct interaction between cholesterol located in the inner lipid layer of the plasma membrane and EGFR has been demonstrated, leading to enhanced receptor dimerization and facilitating downstream signaling in the presence of EGF (Kim et al., 2025).

In this study we showed that NPC1 downregulation or inhibition of its cholesterol transport activity had a major impact on the EGFR lifespan, promoting relocation of the receptor from the cell surface to the late endosomes and lysosomes. A similar behavior has been noted for the SARS-CoV-2 receptor, ACE2, during treatment with U18666A. The plasma membrane depletion of ACE2 is accompanied by its accumulation in autophagosomal/lysosomal compartments, conferring protection of cells against SARS-CoV-2 infection and promoting NPC1 as a promising host-directed antiviral target (La Rosa et al., 2024). Interestingly, a direct interaction between NPC1 and SARS-CoV-2 spike protein that is critical for efficient viral infection has also been demonstrated, pointing to a role for NPC1 beyond its function in cholesterol transport (Khan et al., 2024). On the other hand, cell treatment with cholesterol induces NPC1 downregulation accompanied by ACE2 lysosomal escape, suggesting that NPC1 anchors ACE2 within lysosomes to control the physiological expression of the receptor at the cell surface (Zhang et al., 2025). These data add yet another layer of complexity to the regulatory activity of NPC1 along the endocytic pathway, supporting the multiple roles played by NTCP in the life-cycle of unrelated viruses that either exploit the endocytic machinery to infect the target cells or induce extensive membrane rearrangements to facilitate their replication (Ahmad et al., 2023).

EGFR degradation in cells with downregulated/chemically-inhibited NPC1 is a plausible mechanism contributing to the inhibition of the early stages of HBV infection. Indeed, the NTCP function as a viral receptor depends on its interaction with EGFR. The NTCP-EGFR complex facilitates HBV internalization into intracellular vesicles, providing the appropriate acidic environment for membrane fusion and subsequent release of the nucleocapsid into the cytoplasm. Moreover, EGFR inactivation or inhibition of its interaction with NTCP, greatly restrict HBV infection (Iwamoto et al., 2019). Consistent with the role of EGFR in HBV endocytosis, our data indicated a lower level of viral DNA internalized in NPC1-inhibited cells, likely reflecting the reduced EGFR availability at the plasma membrane. The contrasting behavior of the two HBV receptors under the same experimental conditions is intriguing; however, this may be explained by the peculiar plasma membrane localization of EGFR, which, unlike NTCP, is at least transiently recruited in higher-order, cholesterol-rich membrane nanodomains (Abe et al., 2024).

Intriguingly, exogenous EGFR expression in NPC1-inhibited cells had only a modest impact on restoring the HBV infection to the levels observed in untreated cells. This may be related to the significant EGFR degradation observed in the presence of U18666A, despite the large amounts of exogenous receptor supplied by cell transfection, or to HBV infection being affected by other, EGFR-independent mechanisms. Indeed, a recent report has clearly shown that viral particle entrapping in late endosomes is a major mechanism of inhibition of HBV infection in NPC1-depleted cells, while no marked impact on HBV uptake has been observed (He et al., 2026). This discrepancy regarding the HBV internalization could stem from technical differences between experimental procedures, as in our study we used U18666A-treated cells, a much lower HBV dose for infection and extensive washes to remove unbound virus particles from the cell surface, which may have increased the resolution of the experiment. Nevertheless, in line with the previous report (He et al., 2026), our data indicated that both NPC1 depletion and chemical inhibition of the cholesterol transporter activity had a significant impact on the early stages of the HBV infection while neither viral replication, nor assembly and secretion were affected. Considering the modest rescue of HBV infection by exogenous EGFR expression in NPC1-inhibited cells, it is plausible that EGFR degradation remains a subtle, yet distinct mechanism contributing to the inhibition of HBV infection due to altered cholesterol homeostasis in late endosomes.

Importantly, a series of clinically approved drugs that bind directly to NPC1 or determine the lysosomal cholesterol accumulation characteristic of the NPC1-like phenotype, through other mechanisms (Ahmad et al., 2023), were also potent inhibitors of HBV infection. This shows that NPC1 is a viable therapeutic target for the development of new antivirals against HBV and that drug repurposing may be an efficient strategy to accelerate this process. The antiviral effect of cholesterol homeostasis inhibitors may be even more relevant under the physiological conditions of HBV infection, in the light of the recent findings indicating that HBV hijacks the reverse cholesterol transport pathway of macrophages to efficiently reach the liver (Esser et al., 2023). Because inhibition of HBV entry reduces the nuclear accumulation of cccDNA (Tu and Urban, 2018), using combinations of entry inhibitors may be an efficient approach to prevent HBV vertical transmission or re-infection of patients after liver transplants (Gad et al., 2022). Certainly, these inhibitors deserve further scrutiny *in vivo*, in appropriate animal models of HBV infection, accompanied by comprehensive toxicity studies (Liu et al., 2021).

In addition to the established role in viral infections, emerging data indicate novel NPC1 functions in cancer biology. NPC1 is highly expressed in hepatocellular carcinoma (HCC) cells and tissues, promoting cell survival, proliferation and migration through cholesterol-dependent, independent or dual mechanisms (Li et al., 2025; Deng et al., 2026). Moreover, our study highlights the NPC1 potential as a promising therapeutic target not only for HCC intervention, but also for treatment of other highly aggressive cancers, characterized by strong EGFR overexpression and activation, such as the pancreatic cancer (Oliveira-Cunha et al., 2011) or glioblastoma (Xu et al., 2017).

## 5 Conclusion

Our findings strengthen the concept that NPC1 is an essential pro-viral host factor facilitating the early steps of HBV infection. By adding EGFR to the plethora of proteins regulated by NPC1, with pivotal roles in cancer development, our study extends the therapeutic potential of NPC1 inhibition to treatment of tumors expressing hyper-active forms of EGFR. The discovery of NPC1 inhibitors may thus aid in the pursuit of much needed alternative therapeutic options for patients who develop resistance to current anti-EGFR drugs.

## Data Availability

The datasets presented in this study can be found in online repositories. The names of the repository/repositories and accession number(s) can be found in the article/**Supplementary Material**.

## References

[B1] Abe M. Yanagawa M. Hiroshima M. Kobayashi T. Sako Y. (2024). Bilateral regulation of EGFR activity and local PI(4,5)P(2) dynamics in mammalian cells observed with superresolution microscopy. Elife 13, e101652. doi: 10.7554/elife.101652 39513999 PMC11548882

[B2] Ahmad I. Fatemi S. N. Ghaheri M. Rezvani A. Khezri D. A. Natami M. . (2023). An overview of the role of Niemann-pick C1 (NPC1) in viral infections and inhibition of viral infections through NPC1 inhibitor. Cell. Commun. Signal. 21, 352. doi: 10.1186/s12964-023-01376-x 38098077 PMC10722723

[B3] Hepatitis B. Available online at: https://www.who.int/en/news-room/fact-sheets/detail/hepatitis-b (Accessed May 8, 2026).

[B4] Balmith M. Faya M. Soliman M. E. (2017). Ebola virus: A gap in drug design and discovery - experimental and computational perspective. Chem. Biol. Drug Des. 89, 297–308. doi: 10.1111/cbdd.12870 27637475

[B5] Bremer C. M. Saniewski M. Wend U. C. Torres P. Lelie N. Gerlich W. H. . (2009). Transient occult hepatitis B virus infection in a blood donor with high viremia. Transfusion 49, 1621–1629. doi: 10.1111/j.1537-2995.2009.02188.x 19413737

[B6] Carette J. E. Raaben M. Wong A. C. Herbert A. S. Obernosterer G. Mulherkar N. . (2011). Ebola virus entry requires the cholesterol transporter Niemann-Pick C1. Nature 477, 340–343. doi: 10.1038/nature10348 21866103 PMC3175325

[B7] Deng R. Zheng X. Liu F. Gao J. Wang S. Yun J. . (2026). The NPC1/USP7/p53 axis regulates cholesterol and promotes the proliferation of hepatocellular carcinoma. Oncogene 45, 1386–1397. doi: 10.1038/s41388-026-03739-3 41882104

[B8] Dobrica M. O. Varghese C. S. Harris J. M. Ferguson J. Magri A. Arnold R. . (2024). CTCF regulates hepatitis B virus cccDNA chromatin topology. J. Gen. Virol. 105, 001939. doi: 10.1099/jgv.0.001939 38175123

[B9] Dorobantu C. Macovei A. Lazar C. Dwek R. A. Zitzmann N. Branza-Nichita N. (2011). Cholesterol depletion of hepatoma cells impairs hepatitis B virus envelopment by altering the topology of the large envelope protein. J. Virol. 85, 13373–13383. doi: 10.1128/jvi.05423-11 21994451 PMC3233160

[B10] Eden E. R. Huang F. Sorkin A. Futter C. E. (2012). The role of EGF receptor ubiquitination in regulating its intracellular traffic. Traffic 13, 329–337. doi: 10.1111/j.1600-0854.2011.01305.x 22017370 PMC3261333

[B11] Esser K. Cheng X. Wettengel J. M. Lucifora J. Hansen-Palmus L. Austen K. . (2023). Hepatitis B virus targets lipid transport pathways to infect hepatocytes. Cell. Mol. Gastroenterol. Hepatol. 16, 201–221. doi: 10.1016/j.jcmgh.2023.03.011 37054914 PMC10394270

[B12] Fiorenza M. T. Moro E. Erickson R. P. (2018). The pathogenesis of lysosomal storage disorders: beyond the engorgement of lysosomes to abnormal development and neuroinflammation. Hum. Mol. Genet. 27, R119–R129. doi: 10.1093/hmg/ddy155 29718288

[B13] Gad S. A. Sugiyama M. Tsuge M. Wakae K. Fukano K. Oshima M. . (2022). The kinesin KIF4 mediates HBV/HDV entry through the regulation of surface NTCP localization and can be targeted by RXR agonists *in vitro*. PloS Pathog. 18, e1009983. doi: 10.1371/journal.ppat.1009983 35312737 PMC8970526

[B14] Gehring G. Rohrmann K. Atenchong N. Mittler E. Becker S. Dahlmann F. . (2014). The clinically approved drugs amiodarone, dronedarone and verapamil inhibit filovirus cell entry. J. Antimicrob. Chemother. 69, 2123–2131. doi: 10.1093/jac/dku091 24710028 PMC7110251

[B15] Grinde B. (1982). Selective inhibition of lysosomal protein degradation by the thiol proteinase inhibitors E-64, Ep-459 and Ep-457 in isolated rat hepatocytes. Biochim. Biophys. Acta 701, 328–333. doi: 10.1016/0167-4838(82)90235-7 7039681

[B16] Gripon P. Rumin S. Urban S. Le Seyec J. Glaise D. Cannie I. . (2002). Infection of a human hepatoma cell line by hepatitis B virus. Proc. Natl. Acad. Sci. U.S.A. 99, 15655–15660. doi: 10.1073/pnas.232137699 12432097 PMC137772

[B17] He S. Zhang W. Wang C. Li R. Xu K. Feng B. . (2026). Niemann-Pick C1 maintains cholesterol homeostasis in late endosomes to drive hepatitis B virus endosomal escape. Antiviral Res. 251, 106437. doi: 10.1016/j.antiviral.2026.106437 42140524

[B18] Huang F. Kirkpatrick D. Jiang X. Gygi S. Sorkin A. (2006). Differential regulation of EGF receptor internalization and degradation by multiubiquitination within the kinase domain. Mol. Cell 21, 737–748. doi: 10.1016/j.molcel.2006.02.018 16543144

[B19] Infante R. E. Radhakrishnan A. Abi-Mosleh L. Kinch L. N. Wang M. L. Grishin N. V. . (2008). Purified NPC1 protein: II. Localization of sterol binding to a 240-amino acid soluble luminal loop. J. Biol. Chem. 283, 1064–1075. doi: 10.1074/jbc.M707944200 17989072

[B20] Iwamoto M. Saso W. Nishioka K. Ohashi H. Sugiyama R. Ryo A. . (2020). The machinery for endocytosis of epidermal growth factor receptor coordinates the transport of incoming hepatitis B virus to the endosomal network. J. Biol. Chem. 295, 800–807. doi: 10.1074/jbc.AC119.010366 31836663 PMC6970923

[B21] Iwamoto M. Saso W. Sugiyama R. Ishii K. Ohki M. Nagamori S. . (2019). Epidermal growth factor receptor is a host-entry cofactor triggering hepatitis B virus internalization. Proc. Natl. Acad. Sci. U.S.A. 116, 8487–8492. doi: 10.1073/pnas.1811064116 30952782 PMC6486715

[B22] Kesarwala A. H. Samrakandi M. M. Piwnica-Worms D. (2009). Proteasome inhibition blocks ligand-induced dynamic processing and internalization of epidermal growth factor receptor via altered receptor ubiquitination and phosphorylation. Cancer Res. 69, 976–983. doi: 10.1158/0008-5472.can-08-2938 19176375

[B23] Khan I. Li S. Tao L. Wang C. Ye B. Li H. . (2024). Tubeimosides are pan-coronavirus and filovirus inhibitors that can block their fusion protein binding to Niemann-Pick C1. Nat. Commun. 15, 162. doi: 10.1038/s41467-023-44504-4 38167417 PMC10762260

[B24] Kim D. H. Hong M. T. Bocharov E. V. Park S. Bershatsky Y. V. Volynsky P. E. . (2025). EGFR activation requires cholesterol interaction at the inner leaflet of the plasma membrane. Sci. Adv. 11, eadx2398. doi: 10.1126/sciadv.adx2398 41417903 PMC12716419

[B25] Kwon H. J. Abi-Mosleh L. Wang M. L. Deisenhofer J. Goldstein J. L. Brown M. S. . (2009). Structure of N-terminal domain of NPC1 reveals distinct subdomains for binding and transfer of cholesterol. Cell. 137, 1213–1224. doi: 10.1016/j.cell.2009.03.049 19563754 PMC2739658

[B26] La Rosa P. Tiberi J. Palermo E. Stefanelli R. Tiano S. M. L. Canterini S. . (2024). The inactivation of the Niemann Pick C1 cholesterol transporter restricts SARS-CoV-2 entry into host cells by decreasing ACE2 abundance at the plasma membrane. Cell Biosci. 14, 148. doi: 10.1186/s13578-024-01331-4 39707537 PMC11662611

[B27] Lazar C. Durantel D. Macovei A. Zitzmann N. Zoulim F. Dwek R. A. . (2007). Treatment of hepatitis B virus-infected cells with alpha-glucosidase inhibitors results in production of virions with altered molecular composition and infectivity. Antiviral Res. 76, 30–37. doi: 10.1016/j.antiviral.2007.04.004 17548120

[B28] Li S. Yan L. Li C. Lou L. Cui F. Yang X. . (2025). NPC1 controls TGFBR1 stability in a cholesterol transport-independent manner and promotes hepatocellular carcinoma progression. Nat. Commun. 16, 439. doi: 10.1038/s41467-024-55788-5 39762312 PMC11704005

[B29] Lim S. G. Baumert T. F. Boni C. Gane E. Levrero M. Lok A. S. . (2023). The scientific basis of combination therapy for chronic hepatitis B functional cure. Nat. Rev. Gastroenterol. Hepatol. 20, 238–253. doi: 10.1038/s41575-022-00724-5 36631717

[B30] Liu J. Xiao Q. Xiao J. Niu C. Li Y. Zhang X. . (2022). Wnt/beta catenin signalling: function, biological mechanisms, and therapeutic opportunities. Signal. Transduct Target Ther. 7, 3. doi: 10.1038/s41392-021-00762-6 34980884 PMC8724284

[B31] Liu Y. Maya S. Ploss A. (2021). Animal models of hepatitis B virus infection-success, challenges, and future directions. Viruses 13, 777. doi: 10.3390/v13050777 33924793 PMC8146732

[B32] Lloyd-Evans E. Morgan A. J. He X. Smith D. A. Elliot-Smith E. Sillence D. J. . (2008). Niemann-Pick disease type C1 is a sphingosine storage disease that causes deregulation of lysosomal calcium. Nat. Med. 14, 1247–1255. doi: 10.1038/nm.1876 18953351

[B33] Lok A. S. F. (2024). Toward a functional cure for hepatitis B. Gut Liver 18, 593–601. doi: 10.5009/gnl240023 38533651 PMC11249939

[B34] Lu F. Liang Q. Abi-Mosleh L. Das A. De Brabander J. K. Goldstein J. L. . (2015). Identification of NPC1 as the target of U18666A, an inhibitor of lysosomal cholesterol export and Ebola infection. Elife 4, e12177. doi: 10.7554/eLife.12177 26646182 PMC4718804

[B35] Macovei A. Petrareanu C. Lazar C. Florian P. Branza-Nichita N. (2013). Regulation of hepatitis B virus infection by Rab5, Rab7, and the endolysosomal compartment. J. Virol. 87, 6415–6427. doi: 10.1128/jvi.00393-13 23536683 PMC3648082

[B36] Miura K. Katsuki R. Yoshida S. Ohta R. Tamura T. (2023). Identification of EGF receptor and Thrombospondin-1 as endogenous targets of ER-associated degradation enhancer EDEM1 in HeLa cells. Int. J. Mol. Sci. 24, 12171. doi: 10.3390/ijms241512171 37569550 PMC10418772

[B37] Mosesson Y. Shtiegman K. Katz M. Zwang Y. Vereb G. Szollosi J. . (2003). Endocytosis of receptor tyrosine kinases is driven by monoubiquitylation, not polyubiquitylation. J. Biol. Chem. 278, 21323–21326. doi: 10.1074/jbc.c300096200 12719435

[B38] Oliveira-Cunha M. Newman W. G. Siriwardena A. K. (2011). Epidermal growth factor receptor in pancreatic cancer. Cancers (Basel) 3, 1513–1526. doi: 10.3390/cancers3021513 24212772 PMC3757375

[B39] Orth J. D. Krueger E. W. Weller S. G. McNiven M. A. (2006). A novel endocytic mechanism of epidermal growth factor receptor sequestration and internalization. Cancer Res. 66, 3603–3610. doi: 10.1158/0008-5472.can-05-2916 16585185

[B40] Pantazica A. M. Dobrica M. O. Lazar C. Scurtu C. Tucureanu C. Caras I. . (2022). Efficient cellular and humoral immune response and production of virus-neutralizing antibodies by the Hepatitis B Virus S/preS1(16-42) antigen. Front. Immunol. 13, 941243. doi: 10.3389/fimmu.2022.941243 35935966 PMC9354405

[B41] Pentchev P. G. Comly M. E. Kruth H. S. Vanier M. T. Wenger D. A. Patel S. . (1985). A defect in cholesterol esterification in Niemann-Pick disease (type C) patients. Proc. Natl. Acad. Sci. U.S.A. 82, 8247–8251. doi: 10.1073/pnas.82.23.8247 3865225 PMC391480

[B42] Piccoli E. Nadai M. Caretta C. M. Bergonzini V. Del Vecchio C. Ha H. R. . (2011). Amiodarone impairs trafficking through late endosomes inducing a Niemann-Pick C-like phenotype. Biochem. Pharmacol. 82, 1234–1249. doi: 10.1016/j.bcp.2011.07.090 21878321 PMC7092840

[B43] Pohnl M. Trollmann M. F. W. Bockmann R. A. (2023). Nonuniversal impact of cholesterol on membranes mobility, curvature sensing and elasticity. Nat. Commun. 14, 8038. doi: 10.1038/s41467-023-43892-x 38081812 PMC10713574

[B44] Rus A. A. Militaru I. V. Popa I. Munteanu C. V. A. Sima L. E. Platt N. . (2023). NPC1 plays a role in the trafficking of specific cargo to melanosomes. J. Biol. Chem. 299, 105024. doi: 10.1016/j.jbc.2023.105024 37423302 PMC10407747

[B45] Schloer S. Goretzko J. Kuhnl A. Brunotte L. Ludwig S. Rescher U. (2019). The clinically licensed antifungal drug itraconazole inhibits influenza virus *in vitro* and *in vivo*. Emerg. Microbes Infect. 8, 80–93. doi: 10.1080/22221751.2018.1559709 30866762 PMC6455256

[B46] Shen J. Liu G. Qi H. Xiang X. Shao J. (2023). JMJD5 inhibits lung cancer progression by facilitating EGFR proteasomal degradation. Cell Death Dis. 14, 657. doi: 10.1038/s41419-023-06194-0 37813845 PMC10562424

[B47] Soldati T. Schliwa M. (2006). Powering membrane traffic in endocytosis and recycling. Nat. Rev. Mol. Cell Biol. 7, 897–908. doi: 10.1038/nrm2060 17139330

[B48] Subczynski W. K. Pasenkiewicz-Gierula M. Widomska J. Mainali L. Raguz M. (2017). High cholesterol/low cholesterol: Effects in biological membranes: A review. Cell Biochem. Biophys. 75, 369–385. doi: 10.1007/s12013-017-0792-7 28417231 PMC5645210

[B49] Takayama M. Maeda S. Watanabe D. Takebayashi K. Hiroshima M. Ueda M. (2024). Cholesterol suppresses spontaneous activation of EGFR-mediated signal transduction. Biochem. Biophys. Res. Commun. 704, 149673. doi: 10.1016/j.bbrc.2024.149673 38401305

[B50] Trinh M. N. Lu F. Li X. Das A. Liang Q. De Brabander J. K. . (2017). Triazoles inhibit cholesterol export from lysosomes by binding to NPC1. Proc. Natl. Acad. Sci. U.S.A. 114, 89–94. doi: 10.1073/pnas.1619571114 27994139 PMC5224357

[B51] Tu T. Urban S. (2018). Virus entry and its inhibition to prevent and treat hepatitis B and hepatitis D virus infections. Curr. Opin. Virol. 30, 68–79. doi: 10.1016/j.coviro.2018.04.004 29775812

[B52] Urban S. Bartenschlager R. Kubitz R. Zoulim F. (2014). Strategies to inhibit entry of HBV and HDV into hepatocytes. Gastroenterology 147, 48–64. doi: 10.1053/j.gastro.2014.04.030 24768844

[B53] van Meer G. Voelker D. R. Feigenson G. W. (2008). Membrane lipids: where they are and how they behave. Nat. Rev. Mol. Cell Biol. 9, 112–124. doi: 10.1038/nrm2330 18216768 PMC2642958

[B54] Varghese F. S. Meutiawati F. Teppor M. Jacobs S. de Keyzer C. Taskopru E. . (2022). Posaconazole inhibits multiple steps of the alphavirus replication cycle. Antiviral Res. 197, 105223. doi: 10.1016/j.antiviral.2021.105223 34856248

[B55] Xu H. Zong H. Ma C. Ming X. Shang M. Li K. . (2017). Epidermal growth factor receptor in glioblastoma. Oncol. Lett. 14, 512–516. doi: 10.3892/ol.2017.6221 28693199 PMC5494611

[B56] Yan H. Zhong G. Xu G. He W. Jing Z. Gao Z. . (2012). Sodium taurocholate cotransporting polypeptide is a functional receptor for human hepatitis B and D virus. Elife 1, e00049. doi: 10.7554/elife.00049 23150796 PMC3485615

[B57] Zhang C. Yang M. An Y. Liu M. Li H. Zhao R. . (2025). Real-time monitoring of secretory protein traffic for investigating trafficking regulators of ACE2 in living cells. J. Biol. Chem. 301, 110593. doi: 10.1016/j.jbc.2025.110593 40812417 PMC12446517

[B58] Zhen Y. Caprioli R. M. Staros J. V. (2003). Characterization of glycosylation sites of the epidermal growth factor receptor. Biochemistry 42, 5478–5492. doi: 10.1021/bi027101p 12731890 PMC2765783

