# Supplementary material for: Niemann-Pick type C1 protein regulates epidermal growth factor receptor stability in hepatic cells and facilitates the early steps of Hepatitis B virus infection
Source: Front Cell Infect Microbiol. 2026 Aug 20;16:1905331. doi: 10.3389/fcimb.2026.1905331 (PMC13537993; doi:10.3389/fcimb.2026.1905331)
Supplement: Supplementary file 1 [file Table1.docx]

Supplementary Material

**Figure S1. Cell viability in the presence of U18666A.** HepG2-hNTCP cells were treated for 24 h with U18666A at the indicated concentrations. Cell viability was further evaluated by MTS assay. Data are presented as mean ± SD from three independent experiments with two replicates. Statistical significance was determined using one-way ANOVA (ns, not significant).

**Figure S2. U18666A induces cellular cholesterol accumulation.** HepG2-hNTCP cells were seeded onto coverslips then treated with either 30 µM U18666A or DMSO for 24 h. Samples were fixed with 2% PFA and incubated with 100 µg/mL filipin III, for 2 h. Nuclei were stained with DAPI. Images were acquired using Images were acquired using the Zeiss LSM 730 confocal laser scanning microscope (63x oil-immersion objective).

**Figure S3. NPC1 depletion results in decreased expression of the HBV co-receptor, EGFR.** HepG2-hNTCP cells were either subjected to NPC1 silencing for 48 h (left panel) or treated with 30 µM U18666A for 24 h (right panel). Cells treated with control siRNA sequences (siCtrl) or DMSO, were included as controls. NTCP and EGFR levels were quantified by cell incubation with corresponding primary antibodies and HRP-conjugated secondary antibodies. The colorimetric signal was measured at 450 nm after addition of TMB. Data are represented as percentages from corresponding controls (mean ± SD, two independent experiments with at least three replicates). Statistical significance was determined using a two-tailed Student’s t-test (***p<0.001, ****p < 0.0001).

**Figure S4. Pharmacological inhibition of NPC1 has no effect on NTCP and EGFR mRNA levels.** HepG2-hNTCP cells were treated with either 30 μM U18666A or DMSO for 24 h, followed by mRNA extraction and quantification by RT-PCR. Data are presented as mean ± SD from two independent experiments performed in at least three biological replicates. Statistical significance was determined using a two-tailed Student’s t-test (ns, not significant).

**Figure S5. Quantification of EGFR levels in NPC1- depleted/inhibited cells.** EGFR levels shown in Figure 5B (A) and 5C (B) were quantified by densitometry analysis and normalized to calnexin expression. Data is shown as EGFR levels relative to the corresponding control condition (mean ±SD from three independent experiments). Statistical analysis was performed using a two-tailed Student’s t-test (*p<0.05, ***p<0.001).

**Figure S6. Quantification of EGFR co-localization with CD63 in NPC1-inhibited cells**. The fraction of the EGFR signal overlapping with that of CD63 was quantified in HepG2-NTCP cells treated with DMSO or 30µM U18666A, by measuring the Mander’s M1 co-localization coefﬁcient. Each dot represents an individual cell (n=40 cells per condition). Results from two independent experiments are shown. Statistical significance was performed using a two-tailed Student’s t-test (****p < 0.0001).

**Figure S7. Exogenously expressed EGFR undergoes degradation in NPC1-inhibited cells and promotes a modest rescue of HBV infection.** HepG2-hNTCP were transfected with 1 or 2 µg/ml of PWPXLD-EGFR-WT for 6 h, then treated with 30 µM U18666A or DMSO for 24 h prior to HBV infection. (A-C) EGFR expression was determined by Western blot at 24 h post-drug treatment, by using anti-EGFR (A, C, total EGFR) or anti-HA antibodies (B, exogenously expressed EGFR-HA). EGFR levels were quantified by densitometry analysis, normalized to calnexin expression and shown as relative to the corresponding control condition (mean ±SD from three independent experiments). (C) Cell lysates were treated with PNGase F prior to Western blot detection. (D) Secretion of HBeAg in cell supernatants was quantified by ELISA, at 7 days post- HBV infection. Cells treated with 1 µM Myrcludex B for 3 h prior to viral inoculation were also included as control. Statistical analysis of all data was performed by using a two-tailed Student’s t-test (*p<0.05, **p<0.01, ns, not significant).
